# Characterization of Nanoparticles in Mixtures by Taylor Dispersion Analysis Hyphenated to Inductively Coupled Plasma Mass Spectrometry

**DOI:** 10.1021/acs.analchem.4c00586

**Published:** 2024-03-26

**Authors:** Daniel Baron, Tomáš Pluháček, Jan Petr

**Affiliations:** Department of Analytical Chemistry, Faculty of Science, Palacký University Olomouc, 17. Listopadu 12, 77146 Olomouc, Czech Republic

## Abstract

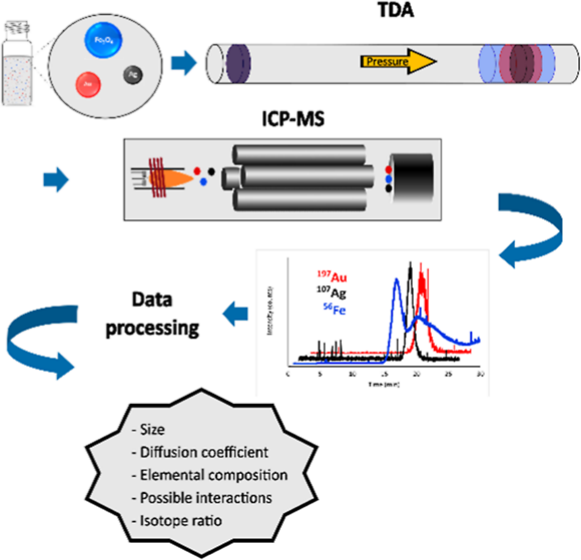

A novel methodology for investigating the behavior of nanoparticles in their mixtures in aqueous high-ionic strength conditions is presented in this work. Our approach utilizes Taylor dispersion analysis in capillaries connected to inductively coupled plasma mass spectrometry (ICP-MS) to probe metal-derived nanoparticles. This methodology simultaneously distinguishes between different kinds of nanoparticles and accurately determines their essential parameters, such as hydrodynamic size, diffusion coefficient, and elemental composition. Moreover, the isotope-specific ICP-MS detection allows for unique targeting of the fate of isotopically enriched nanoparticles. The complexity of our methodology opens the way for studying barely explored areas of interparticle interactions or unequivocal characterization of one type of nanoparticle in complex mixtures without any need for calibration as well as labor-consuming sample preparation.

## Introduction

The last few decades have witnessed tremendous progress in the design of metallic nanoparticles (NPs) and ultrasmall NPs for various applications, such as medicine, sensing, biotechnology, water and sewage treatment, construction, paints, cosmetics, electronics, and energy storage.^1,2^ Interestingly, our understanding of the self-assembly process of NPs and their interactions with other NPs is still quite limited. It is known that interparticle forces among building blocks play a vital role in constructing assembled superstructures; however, the evolution of this process has not been explored yet.^3^ Moreover, introducing micro- and nanosystems into complex real-life conditions, such as living bodies or the environment, could have other important effects on their overall assembled structure. It appears that NP–NP interactions play a crucial role in all the processes and applications described.^4,5^ However, there is a lack of analytical techniques that allow for the simultaneous characterization of freely moving NPs, and their mutual interactions under high ionic strength conditions mimic the real-life situation in living bodies or the environment.

Recently, only a few studies dealing with the behavior of NPs in the presence of other NPs have been published. Pyrgiotakis et al.^6^ used an atomic force microscopy (AFM)-based platform to investigate the NP–NP interaction in biological media by employing NP-modified AFM tips. However, the Fe_2_O_3_, CeO_2_, and SiO_2_ NPs were attached on the AFM tip, and they were not freely moving. Hence, the results only partially reflect the situation in living entities. Riedesel et al.^7^ used titration under UV–vis, while Kaur et al.^8,9^ implemented other “traditional” techniques such as transmission electron microscopy (TEM) or dynamic light scattering (DLS) to study the NP–NP interaction with surfactants. In both papers, NPs were freely moving in solution but could not be distinguished. Therefore, the results cannot be attributed to specific types of analyzed NPs.

Nowadays, Taylor dispersion analysis (TDA) is gaining more attention for the characterization of NPs. TDA is an absolute method for determining the diffusion coefficient of a given analyte from a very small sample volume (subnanoliters). Therefore, it does not require calibration or prior knowledge of the sample concentration. The hydrodynamic radius and size distribution of a given NP population can be calculated using the Stokes–Einstein formula.^10,11^ TDA can be considered the mathematical framework for the analysis of dispersion in solutions using a parabolic velocity profile of pressure-driven laminar flow in a cylindrical tube such as a fused silica capillary. When a sample plug is introduced into the flow, it spreads axially due to convection and diffusion. The longitudinal diffusion is negligible when conducted under well-controlled conditions; thus, the broadening of an analyte plug relates to the diffusion coefficient.^12^ Moreover, TDA measurements do not require any special instrumentation. They can be conducted, for example, on a standard capillary electrophoresis (CE) instrument. In cases where TDA analyses are performed in a buffered environment, they effectively reflect the behavior of the analyte in terms of changes in size and possible interactions with species present in living bodies or the environment.^13−15^

Sensitive inductively coupled plasma mass spectrometry (ICP-MS) detection can transform TDA into a tool that is specifically designed for probing the size of NPs and even ultrasmall NPs in real samples, such as cells, tissues, air, water, and soil, where various NPs from different sources may be present simultaneously. So far, the TDA-ICP-MS approach has been employed to characterize only a specific type of NPs. Labied et al.^16^ demonstrated that the TDA-ICP-MS approach can precisely determine the radius of ultrasmall Gd-containing NPs in various media. The data were also compared with UV detection, although only under specific conditions. Subsequently, the same group investigated Gd release from Gd-chelated polysiloxane NPs.^17^ Finally, Degasperi et al. examined the radius of core–shell gold/silica NPs interacting with proteins, namely, bovine serum albumin and fetuin.^18^ To the best of our knowledge, there has been no publication describing the characterization of NPs in their liquid mixtures.

In our work, we have developed a novel TDA-ICP-MS method that allows for the simultaneous investigation of several aspects of NPs, including their hydrodynamic diameter, size distribution, concentration, elemental composition, isotope ratio, and behavior in the presence of other NPs. These investigations are carried out under conditions that closely resemble real-life scenarios in cells and the environment. We conducted experiments using carboxylated magnetite (Fe_3_O_4_@COOH) NPs in the presence of gold NPs under various conditions (pH). For the TDA analyses, we utilized the CE-ICP-MS interface that was constructed in our laboratory.^19−21^

## Experimental Section

### Materials

Carboxylated iron oxide (II, III) NPs [25 nm (TEM), 5 mg/mL; Fe_3_O_4_@COOH NPs; product no.: 900042], gold NPs [20 nm (TEM), ∼6.54 × 10^11^ particles/mL, 0.057 mg/mL determined by solution ICP-MS;^22^ product no.: 741965], silver dispersion NPs [10 nm (TEM), 0.02 mg/mL; product no.: 730785], silver dispersion NPs [20 nm (TEM), 0.02 mg/mL; product no.: 730793], sodium hydroxide solution (50%, EMPROVE bio), phosphoric acid (≥85 wt %, ACS reagent), acetic acid (≥99%, ReagentPlus), citric acid (99%), boric acid (≥99.5%, for electrophoresis), dimethyl sulfoxide (≥99.7%, CHROMASOLV), and aqueous single-element certified reference material Certipur Ag (1000 mg/L) were supplied by Sigma-Aldrich (St. Louis, MO, USA). The aqueous single-element certified reference materials ASTASOL Tb, Bi, and Sc (1000 ± 2 mg/L) were purchased from Analytika, Ltd. (Prague, Czech Republic). Ultrapure water with a resistivity of 18.2 MΩ cm was used for buffer, and sheath liquid preparations were produced by the Milli-Q reference system (Millipore, France).

All of the used running buffers (ionic strength of 40 mM) were prepared by dissolving the corresponding amount of the above-mentioned acids in ultrapure water, and then 50% (w/w) NaOH was added until the desired pH value was reached. The amounts of acids needed for buffer preparation (to match the ionic strength of 40 mM) were calculated using the Peakmaster software.^23,24^

Vials for TDA measurements were filled with buffer solutions with either 10% (v/v) of a suspension of the gold NPs (0.057 mg/mL) or ultrapure water (when blank analyses were performed). The sample consisted of concentrated Fe_3_O_4_@COOH NPs directly pipetted into the sample vial. In the case of size investigation on Au and Ag NPs, these were injected independently or as a mixture (50:50, v/v). Here, the running buffer was not modified (neither H_2_O nor any NPs were added).

### Taylor Dispersion Analysis

All the TDA experiments were conducted on the CE instrument CE7100 by Agilent Technologies (Waldbronn, Germany) equipped with a diode-array detector (DAD). Analyses were carried out in fused silica capillaries (50 μm I.D., effective length of 51.5 cm in the case of TDA-DAD and 70 cm in the case of TDA-ICP-MS) purchased from Molex (Lisle, IL, USA). They were conditioned by flushing with 0.1 M NaOH for 20 min and subsequently rinsed with H_2_O for another 20 min before first use. Between analyses, capillaries were flushed with 0.1 M NaOH for 2 min, deionized water for 2 min, and BGE for 3 min. Flushing was done at a pressure drop of 935 mbar, and samples were introduced into the capillary at 50 mbar for 5 s. Then, the sample zone was pushed through the capillary toward the detector at a pressure drop of 50 mbar at a temperature of 25 °C. The signal of Fe-based NPs was collected by using a DAD under 200 nm. All analyses were performed in three replicates; thus, the results are expressed as the arithmetic mean ± standard deviation. Mathematical procedures are given in the Supporting Information.^25−28^

### TDA-ICP-MS Method

For element-/isotope-specific detection of metal-derived NPs, CE was coupled with an (ORS)-ICP-MS 7700x instrument (Agilent Technologies, Tokyo, Japan) via the in-house CE-ICP-MS interface.^19−21^ Since no electricity is needed during TDA analyses, deionized water with the addition of Sc, Tb, and Bi (10 ng/mL) was used as a sheath liquid to monitor the stability of an aerosol supplied to an ICP-MS instrument. The RSD of the signal intensity for ^45^Sc, ^159^Tb, and ^209^Bi was less than 6%, indicating the stability of the sheath liquid supply. The ICP-MS instrument was tuned daily to achieve the highest S/N ratio for the analyzed elements. Optimized operating conditions for ICP-MS were as follows: an RF power of 1550 W; a plasma gas flow rate of 15.0 L/min; an auxiliary gas flow rate of 0.9 L/min; a nebulizer gas flow rate of 0.75–0.8 L/min; a makeup gas flow rate of 0.3–0.4 L/min; a collision gas He flow rate of 3.0 mL/min (for Fe NPs determination); and a dwell time of 150 ms for ^54^Fe and ^56^Fe; 100 ms for ^107^Ag, ^109^Ag, ^159^Tb, ^197^Au, ^12^C, and ^34^S; and 80 ms for ^45^Sc, ^159^Tb, and ^209^Bi. The signal for the most abundant Fe isotope, ^56^Fe, and signals of ^107^Ag, ^109^Ag, and ^197^Au were used for the nanoparticle size calculations, whereas ^54^Fe was used to prove TDA results for only Fe-containing NPs. On top of that, the monitoring of ^34^S and ^12^C was adopted as tracers of DMSO representing a nonreacting, well-defined compound. The limit of detection (LOD) was calculated as the signal intensity that equaled three times the S/N ratio. All analyses were performed in three replicates; thus, the results are expressed as an arithmetic mean ± standard deviation.

### DLS and Zetametry Measurements

The mean hydrodynamic diameters and zeta potential values were determined by DLS measurements using the Malvern Zetasizer Nano instrument (Malvern Instruments, Worcestershire, UK). NPs were carefully dispersed in the electrolytes and immediately measured. All analyses were performed in three replicates; thus, the results are expressed as an arithmetic mean ± standard deviation.

## Results and Discussion

### TDA-ICP-MS of NPs in NP-Based Media

**Figure 1:**
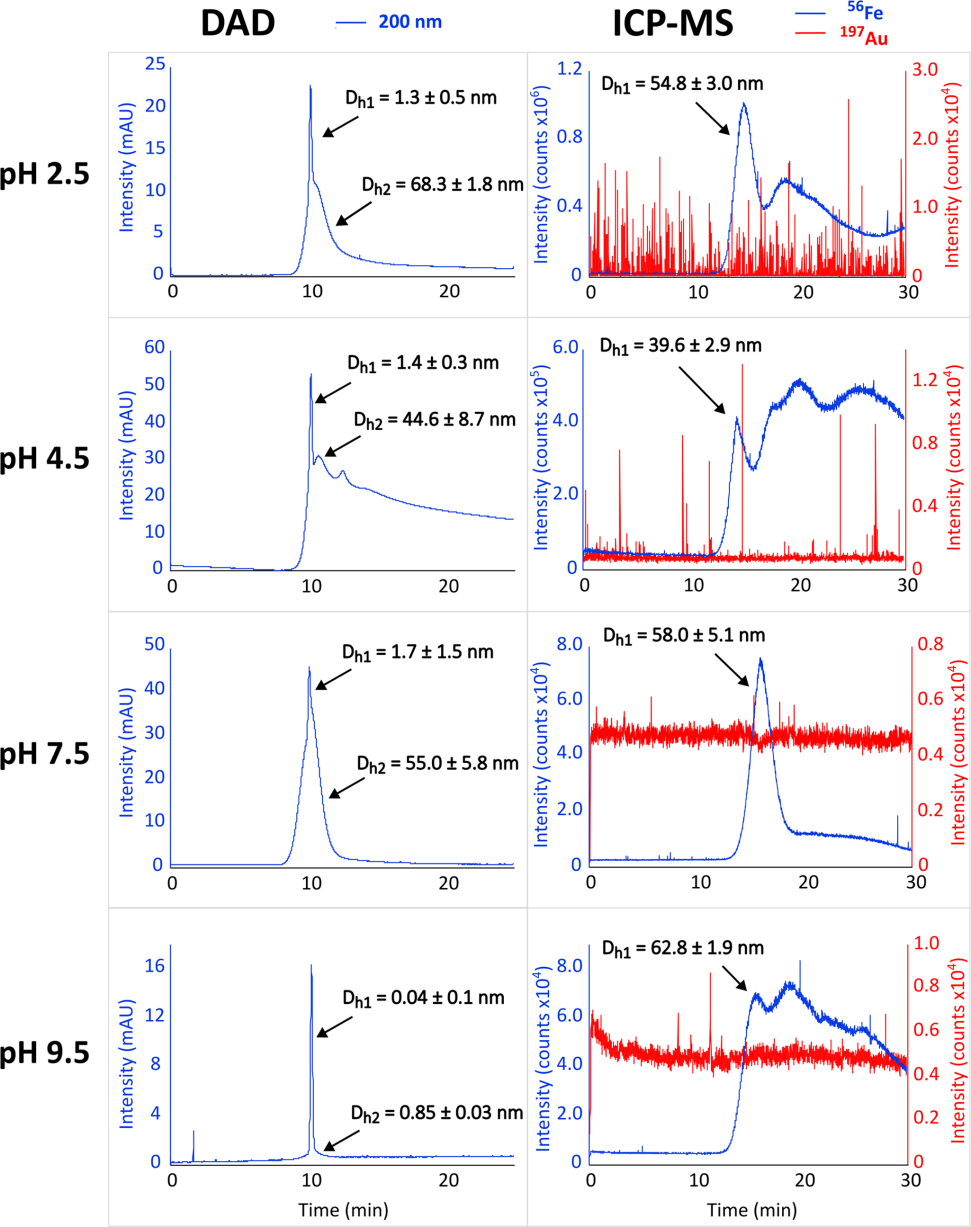
Comparison of the TDA-DAD (left) and TDA-ICP-MS taylorgrams for Fe_3_O_4_@NPs (4.5 mg/mL) in the presence of the Au NPs (TEM 20 nm, 6 μg/mL) at various environments differing in pH.

First, a suspension of 20 nm Au NPs was added to various background media (10%, v/v) with the same ionic strength of 40 mM but varying pH values: 2.5, 4.5, 7.5, and 9.5. Then, a sample of 4.5 mg/mL Fe_3_O_4_@COOH NPs (TEM 25 nm) was subjected to conventional TDA using a DAD and ICP-MS in specific buffers. Data acquired at 200 nm were compared with blank measurements, where 10% ultrapure water was added instead of Au NPs. The resulting taylorgrams are shown in Figure 1. The modified data processing procedure was adopted to obtain a smoothed Gauss function.^29^ The characteristics for the nonaffected NP peak eluting in a void volume were used for the calculations of diffusion coefficients and, subsequently, NPs’ hydrodynamic diameters (see Table 1 and Supporting Information for data processing).

**Table 1:** Comparison of Results Provided by TDA and DLS

pH	sample	hydrodynamic diameter (nm)		
		TDA-DAD^a^^,^^b^	TDA-ICP-MS^c^	DLS
2.5	Fe_3_O_4_@NPs	56.2 ± 12.8	55.1 ± 2.2	67.6 ± 1.5
	Fe_3_O_4_@NPs in Au NPs	68.3 ± 1.8	54.8 ± 3.0	79.0 ± 0.8
	Au NPs	N/A	N/A	85 ± 28^d^
4.5	Fe_3_O_4_@NPs	24.8 ± 7.2	47.6 ± 7.0	94.1 ± 0.9
	Fe_3_O_4_@NPs in Au NPs	44.6 ± 8.7	39.6 ± 2.9	62.2 ± 0.3
	Au NPs	N/A	N/A	117 ± 57^d^
7.5	Fe_3_O_4_@NPs	56.0 ± 2.6	58.5 ± 6.5	57.6 ± 0.1
	Fe_3_O_4_@NPs in Au NPs	55.0 ± 5.8	58.0 ± 5.1	69.9 ± 0.6
	Au NPs	N/A	N/A	31.3 ± 0.1
9.5	Fe_3_O_4_@NPs	0.74 ± 0.1	63.4 ± 6.6	81.6 ± 3.4
	Fe_3_O_4_@NPs in Au NPs	0.85 ± 0.03	62.8 ± 1.9	64.5 ± 1.5
	Au NPs	N/A	N/A	41.9 ± 0.6

a At 200 nm.

b Values in Table 1 stand for *D*_h2_ (see Figure 1).

c At ^56^Fe.

d Irreproducible, possible aggregation.

Both analyzed NPs increased their zeta potential with increasing pH: Fe_3_O_4_@COOH NPs from 3.6 mV at pH 2.5 to −10.6 mV at pH 9.5 and Au NPs from −1.6 mV at pH 2.5 to −22.3 mV at pH 9.5 (see the Supporting Information for more details). As a result, electrostatic repulsion between NPs can be expected only in an alkaline environment. We believe that this could be the reason for the higher fluctuation of the ^197^Au signal at pH 2.5 and 4.5, where NP–NP aggregation might occur.

Furthermore, a significant decrease in the ^197^Au signal was observed at these pH levels, which would be barely possible to discern in a common TDA-DAD. This might be attributed to the adsorption of Au NPs onto the capillary wall. This observation aligns with DLS measurements, where the results for Au NPs at pH 2.5 and 4.5 were not reproducible. The possible aggregation can also lead to an overestimation of the diameter of NPs in the mixture, especially in the case of DAD (as DAD represents a universal detector).

Interestingly, most of the DAD data show partially sharp profiles, resulting in unrealistic NPs’ diameters ranging from 0.04 to 1.7 nm (*D*_h1_). This has been explained by the release of NPs’ stabilizing ligands, which have a higher absorption coefficient than that of the NPs themselves.^30^ The observed sharp peak corresponds to much smaller molecules and not to NPs with a TEM size of 25 nm. On the other hand, the non-Gaussian peaks may be attributed to the partial adsorption of NPs on the capillary wall, NPs’ polydispersity, or open-tube hydrodynamic chromatography.^10,31,32^ In this context, only the use of a specific detection, such as ICP-MS, led to accurate results.

In comparison with traditional DLS, the DLS results cannot be considered reliable since DLS is not element-specific. It can determine only the average size of the given mixture rather than each type of NP independently. The inconsistency with the values declared by TEM (25 nm) is because both DLS and TDA take into account the hydrodynamic diameter in a specific environment, whereas TEM can determine only the core of the studied NPs.

Our TDA-ICP-MS approach can accurately determine the diameter of Fe_3_O_4_@COOH NPs both alone and in the presence of Au NPs, as shown in Table 1. The values observed are consistent at the same pH levels. Variations between electrolytes can be attributed to the different surface chemistry of NPs, including zeta potentials and their hydrodynamic behavior. Furthermore, as indicated by the ICP-MS profiles in Figure 1, multiple peaks were observed, which may be indicative of NPs’ adsorption on the capillary wall or interactions between Fe_3_O_4_@COOH NPs and Au NPs. This complexity also cannot be resolved using conventional TDA-DAD or other measurement techniques, such as DLS or TEM.

### TDA-ICP-MS of NPs’ Mixtures

**Figure 2:**
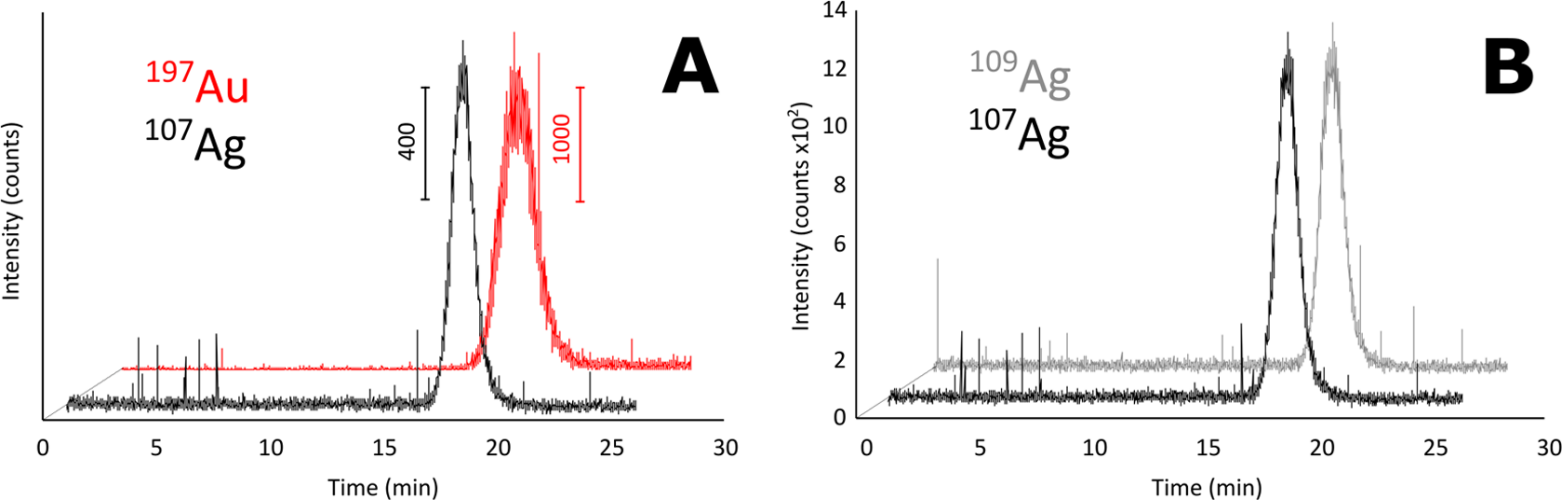
Element-/isotope-specific TDA-ICP-MS. (A) ^107^Ag and ^109^Au taylorgrams showed unequivocal characterization of a mixture of Au (39.4 ± 1.1 nm, 0.03 mg/mL) and Ag NPs (16.5 ± 0.4 nm, 0.01 mg/mL). (B) Simultaneous acquisition of ^107^Ag and ^109^Ag taylorgrams.

The multielemental and isotope-specific character of ICP-MS detection offers another unique capability: the simultaneous TDA sizing of NP mixtures in complex media. In our approach, we injected a mixture of Au and Ag NPs (1:1, v/v) with sizes of 20 and 10 nm (as determined by TEM), respectively, into the capillary and conducted TDA (Figure 2). It is evident that the peak representing Au NPs is broader than the one corresponding to Ag NPs, indicating that Au NPs have a greater diffusion coefficient and thus hydrodynamic diameter. This finding was confirmed by the DLS data obtained from the diluted individual NP samples (Table 2) and the reported sizes of the analyzed NPs.

**Table 2:** Results for Au and Ag NPs Provided by TDA-ICP-MS^a^

sample type	isotope monitored	hydrodynamic diameter (nm)
Au or Ag NPs measured independently	^197^Au	39.0 ± 1.8
	^107^Ag	16.6 ± 0.9
	^109^Ag	16.6 ± 1.3
mixture of Au and Ag NPs	^197^Au	39.4 ± 1.1
	^107^Ag	16.5 ± 0.4
	^109^Ag	16.6 ± 0.3

a In citrate buffer pH 7.0.

The ability to simultaneously determine the NP’s size in complex mixtures is one of the key advantages of our isotope- and element-specific TDA-ICP-MS approach because this task remains challenging for other techniques. Such NPs are often difficult to detect using DAD due to their low absorbance and high scattering. Therefore, conventional TDA-DAD and even DLS provide only an average profile, and other characterization techniques like high-resolution TEM or single-particle ICP-MS are constrained by the environment.

Our TDA-ICP-MS approach is versatile and readily applicable to aqueous and high-ionic-strength environments, e.g., citrate buffer at pH 7.0. Furthermore, the reliability of the calculated hydrodynamic diameters can be confirmed by the analysis of multiple isotopes (not applicable for monoisotopic elements). For example, the results for Ag NPs were confirmed for both isotopes, thus accounting for possible interferences in the setup (Table 2).

Another remarkable feature of the TDA-ICP-MS approach is its ability to determine isotope ratios (see the Supporting Information for procedures^33,34^). The signals obtained for ^107^Ag and ^109^Ag isotopes are shown in Figure 2B. In this case, the ^107^Ag/^109^Ag ratio is 1.0770 ± 0.0046, while the natural ^107^Ag/^109^Ag ratio is 1.0764.^35^ This suggests that commercially available Ag NPs are manufactured from natural Ag sources. This feature of our TDA-ICP-MS approach can be highly beneficial for process control when isotopically enriched NPs are produced or when investigating NPs composed of different isotopes and their fate in the environment or cellular uptake.^36^

Finally, the LOD values were determined to be 37.8 μg/mL for Fe, 0.26 μg/mL for Ag, and 0.24 μg/mL for Au, showcasing the limits of our TDA-ICP-MS approach. These values align with the superior LODs and wide linear dynamic range associated with ICP-MS detection.

## Conclusions

In conclusion, our work has introduced a novel multielemental and isotope-specific TDA-ICP-MS methodology for studying the behavior of NPs in the presence of other NPs with aqueous high-ionic strength conditions that mimic real-life situations in cells and the environment. We conducted experiments with carboxylated magnetite (Fe_3_O_4_@COOH) and Ag NPs in the presence of Au NPs under various conditions, demonstrating the extraordinary potential of our method. We believe that our methodology has wide applicability for addressing numerous questions in the fields of nanomaterial chemistry, nanotoxicology, and medicine, for example, studying the self-assembly processes, effects of different NPs, NPs’ functional agglomerates, and metal-based nanorobots freely moving in living organisms or the environment.

## Supporting Information Available

TDA theory, zeta potentials, taylorgram deconvolution, and isotope ratio analysis (PDF)

## References

[ref1] De Luis B.; Llopis-Lorente A.; Sancenón F.; Martínez-Máñez R. Engineering chemical communication between micro/nanosystems. Chem. Soc. Rev. 2021, 50, 8829–8856. 10.1039/D0CS01048K. 34109333

[ref2] Sajid M.; Plotka-Wasylka J. Nanoparticles: Synthesis, characteristics, and applications in analytical and other sciences. Microchem. J. 2020, 154, 104623 10.1016/j.microc.2020.104623.

[ref3] Li C.; Qin X.; Zhang Z.; Lv Y.; Zhang S.; Fan Y.; Liang S.; Guo B.; Li Z.; Liu Y.; Luo D. Structure-activity collective properties underlying self-assembled superstructures. Nano Today 2022, 42, 101354 10.1016/j.nantod.2021.101354.

[ref4] Guo Y.; Tang N.; Guo J.; Lu L.; Li N.; Hu T.; Zhu Z.; Gao X.; Li X.; Jiang L.; Liang J. The aggregation of natural inorganic colloids in aqueous environment: A review. Chemosphere 2023, 310, 136805 10.1016/j.chemosphere.2022.136805. 36223821

[ref5] Boström M.; Williams D. R. M.; Ninham B. W. Specific Ion Effects: Why DLVO Theory Fails for Biology and Colloid Systems. Phys. Rev. Lett. 2001, 87, 168103 10.1103/PhysRevLett.87.168103. 11690249

[ref6] Pyrgiotakis G.; Blattmann C. O.; Pratsinis S.; Demokritou P. Nanoparticle–Nanoparticle Interactions in Biological Media by Atomic Force Microscopy. Langmuir 2013, 29, 11385–11395. 10.1021/la4019585. 23978039 PMC4438084

[ref7] Riedesel S.; Kaur R.; Bakshi M. S. Distinguishing Nanoparticle–Nanoparticle Interactions between Gold and Silver Nanoparticles Controlled by Gemini Surfactants: Stability of Nanocolloids. J. Phys. Chem. C 2021, 125, 5399–5411. 10.1021/acs.jpcc.1c00220.

[ref8] Kaur R.; Singh K.; Khullar P.; Gupta A.; Ahluwalia G. K.; Bakshi M. S. Applications of Molecular Structural Aspects of Gemini Surfactants in Reducing Nanoparticle–Nanoparticle Interactions. Langmuir 2019, 35, 14929–14938. 10.1021/acs.langmuir.9b02855. 31645104

[ref9] Kaur A.; Sandhu R. K.; Khullar P.; Singh K.; Ahluwalia G. K.; Bakshi M. S. Colloidal Stabilization of Sodium Dilauraminocystine for Selective Nanoparticle–Nanoparticle Interactions: Their Screening and Extraction by Iron Oxide Magnetic Nanoparticles. Langmuir 2021, 37, 6588–6599. 10.1021/acs.langmuir.1c00956. 34015225

[ref10] Moser M. R.; Baker C. A. Taylor dispersion analysis in fused silica capillaries: a tutorial review. Anal. Methods 2021, 13, 2357–2373. 10.1039/D1AY00588J. 33999088

[ref11] Trapiella-Alfonso L.; Ramírez-García G.; d’Orlyé F.; Varenne A. Electromigration separation methodologies for the characterization of nanoparticles and the evaluation of their behaviour in biological systems. TrAC, Trends Anal. Chem. 2016, 84, 121–130. 10.1016/j.trac.2016.04.022.

[ref12] Bello M. S.; Rezzonico R.; Righetti P. G. Use of Taylor-Aris Dispersion for Measurement of a Solute Diffusion Coefficient in Thin Capillaries. Science 1994, 266, 773–776. 10.1126/science.266.5186.773. 17730397

[ref13] d’Orlyé F.; Varenne A.; Gareil P. Determination of nanoparticle diffusion coefficients by Taylor dispersion analysis using a capillary electrophoresis instrument. J. Chromatogr. A 2008, 1204, 226–232. 10.1016/j.chroma.2008.08.008. 18718601

[ref14] Latunde-Dada S.; Bott R.; Hampton K.; Patel J.; Leszczyszyn O. I. Methodologies for the Taylor dispersion analysis for mixtures, aggregates and the mitigation of buffer mismatch effects. Anal. Methods 2015, 7, 10312–10321. 10.1039/C5AY02094H.

[ref15] Gouyon J.; Boudier A.; Barakat F.; Pallotta A.; Clarot I. Taylor dispersion analysis of metallic-based nanoparticles – A short review. Electrophoresis 2022, 43, 2377–2391. 10.1002/elps.202200184. 36153831

[ref16] Labied L.; Rocchi P.; Doussineau T.; Randon J.; Tillement O.; Lux F.; Hagege A. Taylor Dispersion Analysis Coupled to Inductively Coupled Plasma-Mass Spectrometry for Ultrasmall Nanoparticle Size Measurement: From Drug Product to Biological Media Studies. Anal. Chem. 2021, 93, 1254–1259. 10.1021/acs.analchem.0c03988. 33372768

[ref17] Labied L.; Rocchi P.; Doussineau T.; Randon J.; Tillement O.; Cottet H.; Lux F.; Hagege A. Biodegradation of metal-based ultra-small nanoparticles: A combined approach using TDA-ICP-MS and CE-ICP-MS. Anal. Chim. Acta 2021, 1185, 339081 10.1016/j.aca.2021.339081. 34711326

[ref18] Degasperi A.; Labied L.; Farre C.; Moreau E.; Martini M.; Chaix C.; Hagege A. Probing the protein corona of gold/silica nanoparticles by Taylor dispersion analysis-ICP-MS. Talanta 2022, 243, 123386 10.1016/j.talanta.2022.123386. 35313133

[ref19] Šebestová A.; Baron D.; Pechancová R.; Pluháček T.; Petr J. Determination of oxaliplatin enantiomers at attomolar levels by capillary electrophoresis connected with inductively coupled plasma mass spectrometry. Talanta 2019, 205, 120151 10.1016/j.talanta.2019.120151. 31450399

[ref20] Baron D.; Rozsypal J.; Michel A.; Secret E.; Siaugue J.-M.; Pluháček T.; Petr J. Study of interactions between carboxylated core shell magnetic nanoparticles and polymyxin B by capillary electrophoresis with inductively coupled plasma mass spectrometry. J. Chromatogr. A 2020, 1609, 460433 10.1016/j.chroma.2019.460433. 31427136

[ref21] Švecová P.; Baron D.; Schug K. A.; Pluháček T.; Petr J. Ultra-trace determination of oxaliplatin impurities by sweeping-MEKC-ICP-MS. Microchem. J. 2022, 172, 106967 10.1016/j.microc.2021.106967.

[ref22] Ostruszka R.; Půlpánová D.; Pluháček T.; Tomanec O.; Novák P.; Jirák D.; Šišková K. Facile One-Pot Green Synthesis of Magneto-Luminescent Bimetallic Nanocomposites with Potential as Dual Imaging Agent. Nanomaterials 2023, 13, 1027 10.3390/nano13061027. 36985921 PMC10054767

[ref23] Gaš B. PeakMaster and Simul – Software tools for mastering electrophoresis. TrAC, Trends Anal. Chem. 2023, 165, 117134 10.1016/j.trac.2023.117134.

[ref24] https://web.natur.cuni.cz/gas/peakmaster.html (accessed Sept 15, 2023).

[ref25] Taylor G. Dispersion of Soluble Matter in Solvent Flowing Slowly through a Tube. Proc. R. Soc. London, Ser. A 1953, 219, 186–203. 10.1098/rspa.1953.0139.

[ref26] Aris R. On the Dispersion of a Solute in a Fluid Flowing through a Tube. Proc. R. Soc. London, Ser. A 1956, 235, 67–77. 10.1098/rspa.1956.0065.

[ref27] Sharma U.; Gleason N. J.; Carbeck J. D. Diffusivity of Solutes Measured in Glass Capillaries Using Taylor’s Analysis of Dispersion and a Commercial CE Instrument. Anal. Chem. 2005, 77, 806–813. 10.1021/ac048846z. 15679347

[ref28] Cottet H.; Biron J.-P.; Martin M. Taylor Dispersion Analysis of Mixtures. Anal. Chem. 2007, 79, 9066–9073. 10.1021/ac071018w. 17958398

[ref29] Oukacine F.; Geze A.; Choisnard L.; Putaux J. L.; Stahl J. P.; Peyrin E. Inline Coupling of Electrokinetic Preconcentration Method to Taylor Dispersion Analysis for Size-Based Characterization of Low-UV-Absorbing Nanoparticles. Anal. Chem. 2018, 90, 2493–2500. 10.1021/acs.analchem.7b03344. 29359557

[ref30] Höldrich M.; Liu S.; Epe M.; Lämmerhofer M. Taylor dispersion analysis, resonant mass measurement and bioactivity of pepsin-coated gold nanoparticles. Talanta 2017, 167, 67–74. 10.1016/j.talanta.2017.02.010. 28340777

[ref31] Striegel A. M. Hydrodynamic chromatography: packed columns, multiple detectors, and microcapillaries. Anal. Bioanal. Chem. 2012, 402, 77–81. 10.1007/s00216-011-5334-3. 21901463

[ref32] Balog S.; Urban D. A.; Milosevic A. M.; Crippa F.; Rothen-Rutishauser B.; Petri-Fink A. Taylor dispersion of nanoparticles. J. Nanopart. Res. 2017, 19, 287 10.1007/s11051-017-3987-3.

[ref33] Dronov M.; Schram J. A method for increasing the precision of isotope ratio analysis on a Quadrupole ICP-MS based on measurements of lead and strontium. J. Anal. At. Spectrom. 2013, 28, 1796–1803. 10.1039/c3ja50096a.

[ref34] Ponzevera E.; Quétel C. R.; Berglund M.; Taylor P. D. P.; Evans P.; Loss R. D.; Fortunato G. Mass discrimination during MC-ICPMS isotopic ratio measurements: Investigation by means of synthetic isotopic mixtures (IRMM-007 series) and application to the calibration of natural-like zinc materials (including IRMM-3702 and IRMM-651). J. Am. Soc. Mass Spectrom. 2006, 17, 1413–1427. 10.1016/j.jasms.2006.06.001. 16876428

[ref35] Chugaev A. V.; Chernyshev I. V. High-noble measurement of 107Ag/109Ag in native silver and gold by multicollector inductively coupled plasma mass spectrometry (MC-ICP-MS). Geochem. Int. 2012, 50, 899–910. 10.1134/S0016702912110055.

[ref36] Laycock A.; Stolpe B.; Römer I.; Dybowska A.; Valsami-Jones E.; Lead J. R.; Rehkämper M. Synthesis and characterization of isotopically labeled silver nanoparticles for tracing studies. Environ. Sci.: Nano 2014, 1, 271–283. 10.1039/C3EN00100H.

