# Supplementary material for: Characterization of Nanoparticles in Mixtures by Taylor Dispersion Analysis Hyphenated to Inductively Coupled Plasma Mass Spectrometry
Source: Anal Chem. 2024 Mar 26;96(14):5658–63. doi: 10.1021/acs.analchem.4c00586 (PMC11007675; doi:10.1021/acs.analchem.4c00586)
Supplement: Supplementary file 1 — ac4c00586_si_001.pdf [file ac4c00586_si_001.pdf]

Supporting Information

Characterization of Nanoparticles in Mixtures by Taylor Dispersion Analysis Hyphenated to ICP-MS

Daniel Baron, Tomáš Pluháček*, Jan Petr*

Department of Analytical Chemistry, Faculty of Science, Palacký University Olomouc, 17. listopadu 12, 77146 Olomouc, Czech Republic.

Email: tomas.pluhacek@upol.cz (T.P.) and jan.petr@upol.cz (J.P.)

Table of Content	Page
Taylor dispersion analysis theory	S2
Zeta potentials (Table S1)	S3
Taylorgrams deconvolution (Figure S1)	S3
Isotope ratio analysis (Figure S2, Table S2)	S4
ORCID	S6
References	S6

TAYLOR DISPERSION ANALYSIS THEORY

In brief, it is well known that the velocity of a fluid in a cylindrical tube under the steady-state laminar flow varies over the tube cross-section according to a parabolic function of the radius. The dispersion of a solute due to molecular diffusion and convection was analyzed by Taylor.[S1,S2] By assuming the diffusion along the axis of tube negligible, he obtained analytical solutions of the convection-diffusion equations for concentration profiles of fronts (S1) and pulses (S2):

$$\frac{\overline{C}}{\overline{C}_0} = \frac{1}{2} \pm \frac{1}{2} erf\left(\frac{(t - t_R)}{\sigma\sqrt{2}}\right) \qquad \text{(S1)}$$

$$\overline{C} = \frac{M}{2\pi^{3/2} R_C^2 \sqrt{kt}} exp\left(-\frac{(t - t_R)^2}{2\sigma^2}\right) \qquad \text{(S2)}$$

where $\overline{C}$ is the mean solute concentration across the cross-section of the tube, $\overline{C}_0$ is the concentration of the front, t is the time, t_R is the mean residence time, M is the mass of the solute in the pulse, R_C is the tube radius, and σ is the temporal variance of the elution profile related to the dispersion coefficient k and the mean fluid velocity u according to:

$$\sigma^2 = 2\frac{kt_R}{u^2} \qquad \text{(S3)}$$

Aris[S3] extended the Taylor theory by giving an analytical expression for the dispersion coefficient k:

$$k = D + \frac{R_C^2 u^2}{48D} \qquad \text{(S4)}$$

where D is the diffusion coefficient; thus, the diffusion coefficient can be easily determined from this equation using t_R value and σ value obtained by fitting the experimental profiles to eqs. (S1) or (S2). However, the use of eq. (S4) needs to fulfill two conditions: (i) the residence time, which is the ratio of the mean solute residence time to the time required for the solute to diffuse a distance equal to the radius of the capillary, should be greater than the time necessary for a radial concentration non-uniformity to reduce its amplitude by a factor of e (residence time should be much higher than 1.4); and (ii) the Peclet number, which describes the relative rates of mass transfer along the axis of the capillary due to convection and diffusion, should be much higher than 69 (the axial diffusion should be negligible compared to the convection). When these, so-called Taylor's conditions are satisfied, then the diffusion coefficient can be calculated from:

$$D = \frac{R_c^2}{24\sigma^2} t_R \tag{S5}$$

Subsequently, the hydrodynamic radius R_h could be calculated from the Stokes equation:

$$R_h = \frac{k_B T}{6\pi\eta D} \tag{S6}$$

where k_B is the Boltzmann constant, T is the absolute temperature, and η is the solution viscosity.

ZETA POTENTIALS

The zeta potentials were determined for both individual nanoparticle solutions and their mixture (2:1) in ultrapure water, as well as various buffers. The zeta potentials are summarized in Table S1.

Table S1. Comparison of measured zeta potentials

Electrolyte	Fe_3O_4@COOH NPs (mV)	Au NPs (mV)	Mixture of Fe_3O_4@COOH and Au NPs (2:1, v/v), (mV)
ultrapure water	-23.2 ± 3.5	-39.4 ± 3.5	-17.9 ± 2.5
phosphate pH 2.5	3.6 ± 0.5	-1.6 ± 0.6	0.1 ± 0.7
acetate pH 4.5	-0.9 ± 0.3	-8.2 ± 1.8	-4.1 ± 0.4
phosphate pH 7.5	-8.2 ± 4.0	-20.9 ± 1.6	-6.9 ± 0.1
borate pH 9.5	-10.6 ± 1.8	-22.3 ± 1.5	-12.9 ± 1.3

TAYLORGRAMS DECONVOLUTION

The raw TDA-DAD and TDA-ICP-MS Taylorgrams suffer from inaccuracies in determining peak apex position and peak width due to the sequential nature of signal acquisition and deviations from the Gaussian

curve. To address this, all Taylorgrams were smoothed, and those with non-Gaussian complex traces were deconvoluted to extract individual Gaussian contributions using OriginPro software (version 2020).

The hydrodynamic diameters were calculated only for the first eluting peak, representing a fraction of NPs unaffected by unwanted adsorption or separation effects that broaden peak width. The position of the peak apex for the non-affected NPs peak was verified using TDA analysis of DMSO, which does not interact with either NPs or the capillary wall itself (Fig. S1A). The correct input FWHM and peak apex values were then converted to hydrodynamic diameters (diffusion coefficients) using the mathematical methods described in part S2. The representative output is depicted in Fig. S1B.

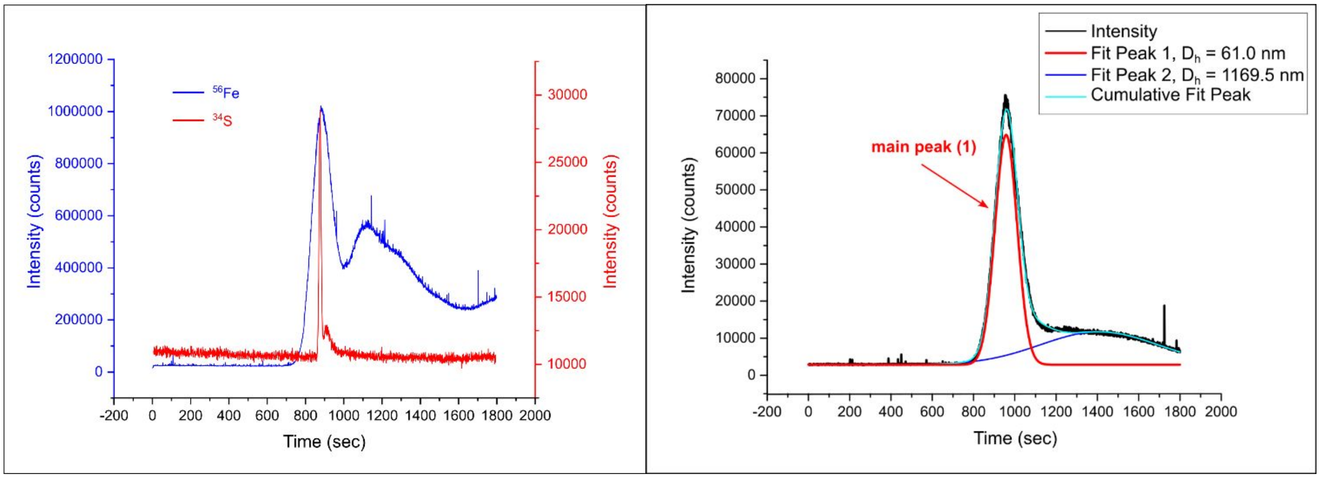

Figure S1. Assessment of a non-altered NPs' peak position in the Taylorgram using DMSO analysis under identical conditions (left); the deconvolution of the ^{56}Fe TDA-ICP-MS cumulative Taylorgram for Fe_3O_4@NPs in the presence of Au nanoparticles, at a buffer pH of 2.5 (left) and 7.5 (right). The deconvolution process reveals a main peak corresponding to the NPs with a hydrodynamic diameter of 61.0 nm (right).

ISOTOPE RATIO ANALYSIS

The deconvolution of the Taylorgrams enables the determination of the NPs' isotope ratios, following a procedure introduced by Dronov and Shram[S4] for accurate isotope ratio assessment using conventional quadrupole ICP-MS

The isotope ratio $r_{(i/j)}$ is defined as per equation (S7):

$$\frac{A(^iI)}{A(^jI)} = \frac{n(^iI)}{n(^jI)} = r_{(i/j)} \quad \text{(S7)}$$

where $A(^iI)$ and $A(^jI)$ are the peak areas of the measured isotopes i and j of NPs derived element; $n(^iI)$ and $n(^jI)$ represent the molar quantity of the isotopes. Thus, the measured isotope ratio ($r_{(i/j)}$) is derived from the integration of smoothed Taylorgram curves.

Several key conditions contribute to obtaining isotope ratios with acceptable measurement uncertainty. First, smoothing Gaussian curves for both studied isotopes is crucial (Fig. S2). Second, the isotope ratio is calculated using peak areas. Finally, a suitable isotope ratio certified reference material (CRM) is utilized for mass discrimination and instrument drift correction.

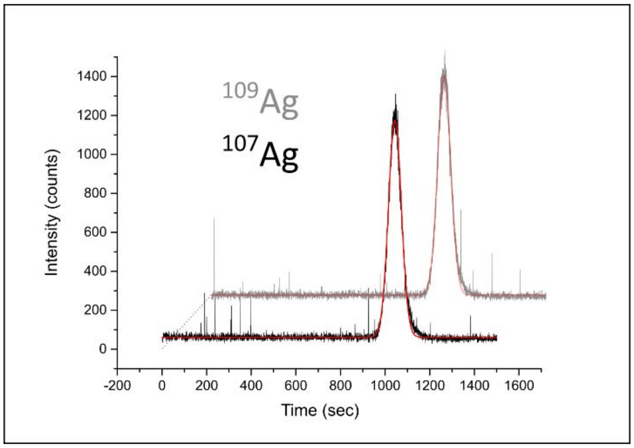

Figure S2. The example of the smoothed Taylorgrams for both silver isotopes.

The corrected isotope ratio $R_{(i/j)}$ is determined through equation (S8):

$$r_{(i/j)}\,K = R_{(i/j)} \quad \text{(S8)}$$

Here, *K* represents a simple mass discrimination factor calculated as the ratio between the theoretical and measured isotope ratio for a suitable CRM diluted at a similar concentration as the NPs solution.[S5] For the isotope analyses of $^{109}Ag/^{107}Ag$ using our setting, the calculated correction factor is 1.041.

While our approach facilitates isotope ratio estimation, it does not aim for highly accurate absolute isotope ratio measures achievable with techniques such as TIMS or multicollector ICP-MS. Nevertheless, the

combination of peak smoothing and summing the area under the NPs curve effectively compensates for the sequential nature of quadrupole ICP-MS monitoring, resulting in reliable isotope ratios (Table S2). The proposed isotope analysis demonstrates acceptable precision of ≤2% and an accuracy (recovery) of approximately 96% without correction and 101% with a correction. Moreover, the TDA-ICP-MS approach can utilize commercially available isotope CRMs without restrictions on analyte form/specie.

Table S2. Isotope ratio estimation for natural Ag NPs (10 nm and 20 nm, TEM)

Sample, mass discrimination factor applied	Isotope ratio	Precision (RSD, %)	Accuracy (recovery, %)
Ag NP 10 nm, without correction	1.0467 ± 0.0213	2	97
Ag NP 10, with correction	1.0896 ± 0.0221	2	101
Ag NP 20 nm, without correction	1.0342 ± 0.0045	0.4	96
Ag NP 20, with correction	1.0767 ± 0.0046	0.4	100

ORCID

Daniel Baron 0000-0003-1607-2458

Tomáš Pluháček 0000-0002-7417-0016

Jan Petr 0000-0002-9961-6459

REFERENCES

(S1) Moser, M. R.; Baker, C. A.; *Analytical Methods* **2021**, *13*, 2357–2373.

(S2) Taylor, G. *Proceedings of the Royal Society A* **1953**, *219*, 186–203.

(S3) Aris, R. *Proceedings of the Royal Society A* **1956**, *235*, 67–77.

(S4) Dronov, M.; Schram, J. *Journal of Analytical Atomic Spectrometry* **2013**, *28*, 1796–1803.

(S5) Ponzevera, E.; Quétel, C. R.; Berglund, M.; Taylor, P. D. P.; Evans, P.; Loss, R. D.; Fortunato, G. *Journal of the American Society for Mass Spectrometry* **2006**, *17*, 1413–1427.
